# Evaluation of Interventions to Address Moral Distress: A Multi-method Approach

**DOI:** 10.1007/s10730-023-09508-z

**Published:** 2023-07-10

**Authors:** Lucia D. Wocial, Genina Miller, Kianna Montz, Michelle LaPradd, James E. Slaven

**Affiliations:** 1 Medstar Washington Hospital Center, John J. Lynch, MD Center for Ethics, 110 Irving Street, NW EB 310, Washington, DC 20002 USA; 2 Fairbanks Center for Medical Ethics, Charles Warren, Indianapolis, IN USA; 3 Indiana University Health, Indianapolis, IN USA; 4 Syneos Health, Morrisville, NC USA; 5 Department of Biostatistics and Health Data Science, Indiana University, Bloomington, IN USA

**Keywords:** Moral distress, Moral agency, Moral community, Ethics discussions

## Abstract

Moral distress is a well-documented phenomenon for health care providers (HCPs). Exploring HCPs’ perceptions of participation in moral distress interventions using qualitative and quantitative methods enhances understanding of intervention effectiveness. The purpose of this study was to measure and describe the impact of a two-phased intervention on participants’ moral distress. Using a cross-over design, the project aimed to determine if the intervention would decrease moral distress, enhance moral agency, and improve perceptions about the work environment. We used quantitative instruments and explored participants’ perceptions of the intervention using semi-structured interviews. Participants were from inpatient settings, within three major hospitals of a large, urban healthcare system in the Midwest, United States. Participants included nurses (80.6%) and other clinical care providers. Using generalized linear mixed modeling we assessed the change in each of the outcome variables over time controlling for groups. Interviews were audiotaped and professionally transcribed. The written narratives were coded into themes. The change in scores on study instruments trended in the desired direction however did not meet statistical significance. Qualitative interviews revealed that intervention effectiveness was derived from a combination of learning benefits, psychological benefits, and building community that promoted moral agency. Findings demonstrate a clear link between moral distress and moral agency and suggest that Facilitated Ethics Conversations can enhance the work environment. Findings provide insight for developing evidenced-based approaches to address moral distress of hospital nurses.

Moral distress is a growing problem for health care providers in general (Allen et al., 2013; Houston et al., 2013; Whitehead et al., 2015) and is a powerful impediment to ethical practice (Huffman & Rittenmeyer, 2012; Piers et al., 2011). Associated organizational problems include poor-quality patient care, patient safety issues, and decreased patient satisfaction (Morley et al., 2017). The concept of moral distress has been studied predominantly in professional nursing yet is established as an interprofessional phenomena that potentially impacts all bedside clinicians (Hamric & Epstein, 2017; Sanderson et al., 2019). Moral distress has been linked to burnout and intention to leave one’s position (Emple et al., 2021; Henrich et al., 2017; McAndrew et al., 2016; Whittaker et al., 2018). Multiple studies report the negative impact of high levels of moral distress on clinicians (Austin et al., 2017; Wiegand & Funk, 2012; Wilson et al., 2013) and patients (Henrich et al., 2017; Piers et al., 2011; Ulrich et al., 2019). The wide variability reported in the literature regarding what contributes to and is associated with moral distress suggests a robust environmental component specific to the area where people work (DeVeer et al., 2013; Huffman & Rittenmeyer, 2012; McAndrew et al., 2018; Sauerland et al., 2015). DeVeer et al. (2013) advocate for measuring, tracking, and reporting in real time HCP levels of moral distress so that it can be addressed. The widespread experience of nurses’ moral distress in relation to the COVID 19 pandemic suggests that to retain nurses in the workforce, we must invest in interventions that address moral distress when it happens (Simonovich et al., 2022). This manuscript reports the findings of a multi methods research study aimed at reducing moral distress, enhancing moral agency and improving the ethical climate.

## Background

Moral distress occurs when a health care provider feels seriously compromised as a moral agent in practicing under accepted professional values and standards (Pauly et al., 2012). Long term moral distress can compromise one’s integrity and ability to deliver effective care (Ulrich et al., 2019), resulting in poor quality patient care (Henrich et al., 2017). The compromise to integrity is one thing that differentiates moral distress from other distress (Berger et al., 2019; Thomas & McCullough, 2018). Because violations of integrity cut at the heart of an individual’s core values, reducing moral distress and the threat to providers’ moral integrity is an essential goal (Halpern, 2011).

Collegial relationships and perceptions of ethical climate significantly influence feelings of moral distress (Atabay et al., 2015; Asgari et al., 2019; DeVeer et al., 2013). In the clinical setting, many barriers prevent individuals from raising ethical concerns or acting on their concerns, including perceptions about the work environment and lack of opportunity or skill in framing the concerns (Musto & Rodney, 2018; Wocial et al., 2010). Empowerment or moral agency, acting on one’s moral judgments, is gaining attention as an area of focus when exploring strategies to address moral distress (Browning, 2013; Carnevale, 2020; Robinson et al., 2014; Traudt et al., 2016).

Recognizing relational agency as a central component to moral action shifts the focus away from interventions that focus solely on the individual to considerations of interventions that impact the individual and the environments in which they work (Musto et al., 2015). When individuals have the opportunity and ability to raise ethical concerns, they feel a sense of power related to their ability to address those concerns and act as effective moral agents (Hamric & Epstein, 2017; Wocial et al., 2010). Using a semi-structured conversation format, a skilled facilitator can create a forum in which health care providers can raise their concerns and discover shared meaning when their values are challenged (Wocial et al., 2010). Informal measurement of moral distress using the Moral Distress Thermometer (MDT) pre and post facilitated ethics discussion suggests they can lower moral distress (pre mean 3.46, post mean 2.79; paired t-test: p = 0.002) (Hamric & Epstein, 2017).

Discussion-based strategies for addressing moral distress have been shown to address emotional responses, build confidence, and promote skill in navigating ethical challenges, interprofessional team collaboration, and assist in managing conflict (Chiafery et al., 2018; Janssens et al., 2015; Hamric & Epstein, 2017). Even so, there is a need for research to further validate the efficacy of this type of approach (Zeydi et al., 2022). Research involving the concept of moral distress has been complicated by lack of consensus on a clear definition and its core components (Kolbe & de Melo-Martin, 2022; Epstein et al., 2019; Sanderson et al., 2019; Morley et al., 2017; Dudzinski, 2016). The following definition of moral distress was used for this research: “moral distress occurs when an individual’s moral integrity is seriously compromised, either because one feels unable to act in accordance with core values and obligations, or attempted actions fail to achieve the desired outcome” (Wocial & Weaver, 2013, 167).

This project evaluated the impact of participation in Facilitated Ethics Conversations (FEC)s on clinicians’ levels of moral distress. In addition to participation in FECs, participants were exposed to public posting of aggregate moral distress scores (including contributing factors as reported by participants). Sharing a visual display of aggregate levels of moral distress is an innovative approach to facilitate an open, transparent atmosphere, serving as one of the hallmarks of an ethical work environment (Hamric & Wocial, 2016). Specifically, the project aimed to determine if participation in FECs would decrease moral distress, enhance moral agency, and improve perceptions about the work environment. Finally, we looked for a relationship between intent to leave and levels of moral distress.

This paper describes clinicians’ perceptions of participation in a two-part intervention that included attending FECs as a strategy for addressing moral distress and exposure to information about aggregate levels of moral distress. Quantitative and qualitative methods were used to provide an in-depth understanding of how the interventions were or were not effective as an intervention for managing moral distress.

## Methods

### Design

**Fig. 1:**
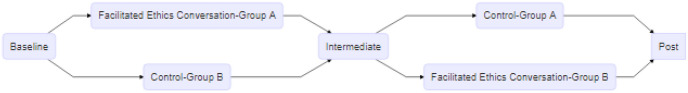
Study design. This figure depicts the flow of interventions and identifies data collection points for survey data. Both groups A and B received the public posting of aggregate moral distress scores and contributing factors at all times during the study period. Group A units received the facilitated ethics conversation with aggregate posting intervention first and only aggregate posting (control) second. Group B received the aggregate posting of scores (control) first and then received the facilitated ethics conversation intervention with aggregate posting of scores

A cross-over design was used to mitigate the impact of confounding variables and reduce the potential for carryover from exposure to the intervention. Participants completed baseline, intermediate, and post intervention instruments, in addition to reporting weekly levels of moral distress and identifying contributing factors. Each intervention phase (aggregate posting with FECs and aggregate posting without FECs) lasted three weeks with a two-week break between interventions. Figure 1 illustrates the framework for the study.

### Procedures

After receiving Institutional Review Board approval, nursing and medical leaders at a large academic health center in the Midwest United States with three hospitals were approached for support of the project. Nursing units were recruited via email to nurse leaders and personal contact with the first author. Commitment to participate in the research evolved over time, meaning nurse leaders did not agree to participate at the same time. Once a nurse leader agreed to support the project, nursing units were randomized, two units assigned to group A (transplant and cardiac surgical ICU) and two units assigned to group B (oncology and bone marrow transplant). Assignment to group A or Group B was based on a coin toss. Two units at a time participated in the intervention phase of data collection. Due to the intensive time commitment of the facilitator, only one unit at a time could receive the FEC intervention. Once one unit was assigned to either group A or B, the other unit participating in the study at the same time was assigned to the unassigned group. Because public posting of aggregate moral distress scores with contributing factors (control group) was new to the unit, there was concern that exposure to this information would influence participant responses. For this reason, the research team used a crossover design.

**Fig. 2:**
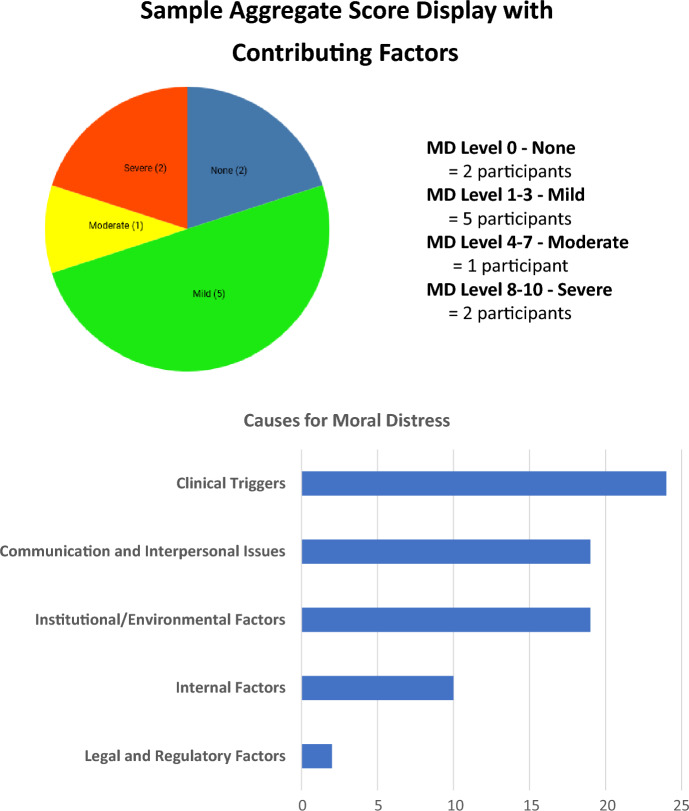
Sample aggregate score display with contributing factors

At baseline, intermediate and post time points (Fig. 1), all participants completed the three study instruments (described below). Once a week, every week during data collection, participants completed the moral distress thermometer, including contributing factors and these results were used to create unit specific aggregate scores that were posted weekly on the nursing unit during quantitative data collection (see Fig. 2). Aggregate scores were created by providing the total number of participants identifying an MDT score in a particular range and totaling the number of identified contributing factors to their moral distress. Additionally, each participant was asked to complete the MDT (no contributing factors) both pre and post FEC.

Qualitative interviews were conducted within three months of completion of quantitative data collection. When completing the final study instruments at the post time point, participants had the opportunity to self-identify if they were interested in participating in semis-structured interviews. Inclusion in the interview portion of the study required participation in moral distress self-scoring and attendance at a minimum of two FECs. There was no attempt to link performance on study instruments with interviews. The first author facilitated all the FECs. The second author conducted all participant interviews and had no prior relationships with the interview participants. The first author did not participate in the interview process or the initial coding process for qualitative data analysis. Both authors are formally trained ethicists and experienced facilitators of ethics discussions.

### Participants

All nurses, social workers, physicians, and other staff (e.g., chaplains, respiratory therapists, music therapists) affiliated with the identified nursing units were eligible to participate in this project. Staff affiliated with participating units were informed of the study first by introduction at regular staff meetings, daily unit huddles, and then via email. The email included details of participation, including incentives for participation and a link to survey instruments. Incentive for participation included individual coffee gift cards for participants in addition to food during the FECs and the opportunity for the nursing unit with the highest participation rate to select a charity to receive a $200.00 donation on behalf of the staff. Requirements for participation included reporting once a week their level of moral distress, identifying factors that contributed to it, and completion of survey instruments at three points during the project (baseline, an intermediate point, and post-intervention phases) and participation in at least two FECs. Enrolled participants received weekly email reminders to report their level of moral distress and emails at baseline, intermediate and post data collection time points with clickable links to complete the survey instruments.

A pediatric oncology unit was the first unit recruited to participate in the study. After completion of data collection for this unit minor modifications to the protocol and quantitative instruments were made. Because of these modifications, the authors determined that the quantitative data would not be comparable to units that participated under the new protocol. Ultimately four additional units (adult cardiac surgical ICU, adult bone marrow transplant ICU, adult oncology, and adult transplant ICU) participated in the project. The units varied in size from 20 to 30 patients with similar staff to patient ratios due to patient acuity. Each of the units had dedicated physician coverage, meaning the nurses and physicians working on the units were well known to each other. The age and levels of experience of the nursing staff was similar across all four units. Because of changes in the protocol and quantitative instruments after the pediatric oncology unit, only data from the four adult units were included in the quantitative analysis of the intervention. Because the qualitative interviews were based on open ended questions about the experience, the interview participants were from all five participating inpatient units.

### Intervention

During the quantitative data collection period, a poster including information about and a list of resources for addressing moral distress was available on each participating nursing unit in staff only areas of the unit. One phase of the study (identified as the control) was the public posting of aggregate moral distress scores with contributing factors. The display included information related to the identified contributing factors and a pie chart showing the distribution of scores based on colors (mild green (0–3), moderate yellow (4–7), severe red (8–10)) (see Fig. 2). The intervention phase included participation in a facilitated ethics conversation (FEC) in addition to exposure to the aggregate moral distress scores and contributing factors.

Time and place for the FECs was determined in collaboration with unit leaders, typically to coincide with a mealtime, since food was provided as part of the incentive to participate. Posters were placed on the unit advertising times for the FECs during the intervention phase. The FEC followed the format and structure described by Helft et al. (2009) and informally utilized mapping strategies described by Dudzinski (2016). The FEC is a discussion format with no set agenda where reflective dialogue and sharing of experiential narratives are encouraged. The facilitator uses various techniques to help participants increase their abilities and confidence in dealing with ethically challenging situations and to provide an environment free of judgment for disclosure and frank discussion of morally troubling situations. During the FEC intervention phases, FECs were available nine times, including both day and night shifts, and on weekends. Unit routine dictated FEC schedules. FEC occurrence was not tied to aggregate measures of moral distress. Finally, participants received a paper version of the instrument to measure moral distress to report levels immediately pre and post participation in the FEC. FECs lasted 30–60 minutes. Food was served during the FEC and duration depended largely on participants’ ability to remain away from patient care. Not infrequently, participants would leave during an FEC and return or leave and not return before the discussion was concluded due to patient care needs.

### Instruments

Instruments for the study were selected for their brevity in part to reduce the response burden of participants. Instruments used for the study are available upon request.

#### Demographic Information

Participants were asked to provide minimal demographic information including their sex, race, ethnicity, age, years in practice, shift worked, and role in patient care. Additionally, they were asked to provide information regarding their experience with ethics training and ethics resources, if they felt it was important to have an opportunity to address ethics challenges, and whether they were considering leaving their current position.

#### Moral Distress Thermometer (MDT)

The moral distress thermometer (MDT) is a numeric instrument with word triggers designed to measure levels of moral distress validated against the Moral Distress Scale-Revised instrument (Wocial & Weaver, 2013). The original instrument defines moral distress, asks respondents to identify if they have it, rate the level of it, and provides a time reference (e.g., within the last two weeks). The MDT was modified for this study to reflect the time frame of data collection. The word triggers were simplified, effectively stratifying the instrument into thirds (mild, moderate, or severe), to support the visual display of results. Reliability testing for the MDT has not been reported, due in part to the challenges of establishing reliability for a single item instrument and the dynamic nature of the experience of moral distress. In addition to the MDT, participants were given a list of contributing factors to identify cases of their moral distress. The list of contributing factors was created from factors identified in the literature as contributing to moral distress (Hamric et al., 2012).

#### Modified Moral Activation Scale (mMAS)

The Nursing Ethical Involvement Scale (NEIS) is a 50-item Likert scale designed to measure nurses’ perceptions of environments in which they practice and their likelihood of taking specific actions in response to a hypothetical ethical challenge. The 16-item nurse moral activism subscale (alpha reliability coefficients 0.83) of the NEIS was used to measure moral agency (Penticuff & Martin, 1987). With permission, we modified the subscale to update language from the original 1987 version (Penticuff, personal communication, 2017).

#### Modified Hospital Ethical Climate Survey (mHECS)

We utilized two of the five sub-scales (peer: alpha reliability coefficients 0.73 and hospital: alpha reliability coefficients 0.77) of the Hospital Ethical Climate Survey to measure perceptions about the environment (Olson, 1998). With permission, the study team updated these subscales both to reflect the shortened version of the HECS and to update the language from the original instrument (Olson, personal communication, 2017).

### Data Analysis

#### Statistical Methods

We compared demographics and outcome variables between groups (participants during the control phase and participants during the intervention phase), using Chi-squared test, Fisher’s exact, Students T-test, or Kruskal Wallis test where appropriate. While the scores themselves were found not to be normal, the change scores were found to be normal. Thus, we utilized generalized linear modeling (GLM) to assess the change in each of the outcome variables over time controlling for group. We calculated least squared means with a Tukey HSD adjustment for the p-value. We analyzed data using SAS v 9.4.

#### Qualitative Methods

A qualitative descriptive approach was used to describe clinician’s perceptions of participation in the study (Sandelowski, 2000). Interviews were used to reveal a deeper understanding of the experience of participating in FECs. Open-ended, semi-structured interviews were conducted using a series of questions and prompts (Questions and prompts provided in Appendix A). All interview participants read the written definition of moral distress used on the Moral Distress Thermometer just prior to the recorded interview. Respondent validation, also known as member checking, was used during the interview process to increase credibility (Henderson & Rheault, 2004). The interviews were audio recorded and transcribed into written text by an independent transcription service.

Interview coding triangulation occurred using three, independent coders to lend credibility, dependability, and confirmability to the study (Henderson & Rheault, 2004). Transcripts were initially coded using a line-by-line code-recode method, then subsequently converted to themes. The data analysis team included the second (GM) and third (KM) authors and a secondary coder. All coders had prior knowledge and experience in qualitative research methods and data analysis. The team met periodically during the coding process to compare and agree on a set of codes and establish a codebook. The first author conducted an audit of all coded transcripts to ensure coding agreement and consistency. Data saturation was reached. Final thematic analysis was conducted by grouping codes into major themes. The primary investigator of the intervention study reviewed a random selection of transcripts and the final code book to lend expertise and confirmability to the findings (Henderson & Rheault, 2004).

## Results

### Participants

**Fig. 3:**
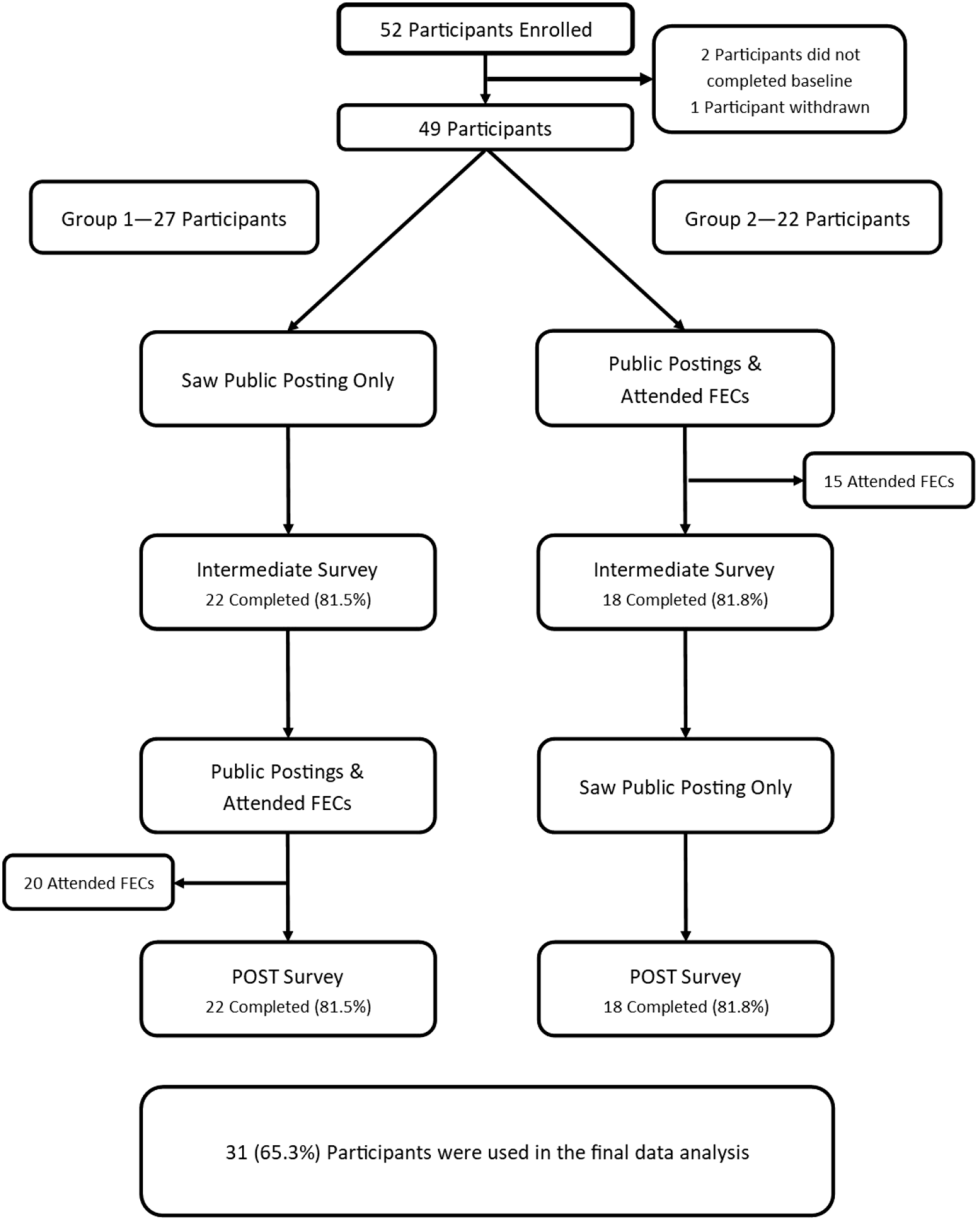
Participant recruitment and progress through the study

Email invitations were sent to 354 individuals, 5 returned not eligible, and 268 did not respond to the invitation. Of the 81 responding potential participants, 29 (35.8%) declined to participate and 52 (64.2%) individuals enrolled in the study. One participant withdrew and only 31 participants completed the necessary surveys to be included in the final quantitative data analysis (72.8% of the original enrolled participants) (See Fig. 3). Table 1 summarizes the demographic data for participants. There were no significant differences in the demographic variables between the groups of participants indicating that groups were similar at baseline. Participants in this study held a nursing role (80.6%) and were primarily white (90.3%) and females (93.5%) which is consistent with the demographics of nurses in the eligible recruitment population. Multiple attempts to recruit physicians and other professions were ultimately unsuccessful, this despite previously mentioned incentives for participation. Interestingly 16.1% of study participants expressed an intent to leave their current position due to moral distress. From the original 52 enrolled participants, 20 self-selected to participate in the qualitative interviews for the study.

**Table 1:** Participant demographics

Variable	Level	*N* (%)^a^
Gender	Male	2 (6.5)
	Female	29 (93.5)
Race	African American	3 (9.7)
	White	28 (90.3)
Ethnicity	Hispanic or Latino	1(3.2)
	Not Hispanic or Latino	28 (90.3)
	Not reported	2 (6.5)
Age	20–30	12 (38.7)
	31–40	6 (19.4)
	41–50	7 (22.5)
	51–60	6 (19.4)
Years in practice	Less than 1	2 (6.5)
	1–2 Years	7 (22.5)
	3–5 Years	6 (18.8)
	6–10 Years	8 (25.8)
	11–15 Years	3 (9.7)
	> 15 years	6 (19.4)
Shift	Days	25 (80.6)
	Nights	6 (19.4)
Role	Nurse	25 (80.6)
	Other	5 (16.1)
	Not Reported	1 (3.2)
Currently considering leaving their current job	Yes	5 (16.1)
Considered leaving:	A little	1 (20)
	Some	3 (60)
	A lot	1 (20)
Importance of Ability to Discuss ethical issues	Somewhat	11 (35.5)
	Very	20 (64.5)

^a^Group A and B were compared, and no statistically significant differences were found in demographics between the two groups

### Quantitative Outcomes

#### Moral Distress

**Fig. 4:**
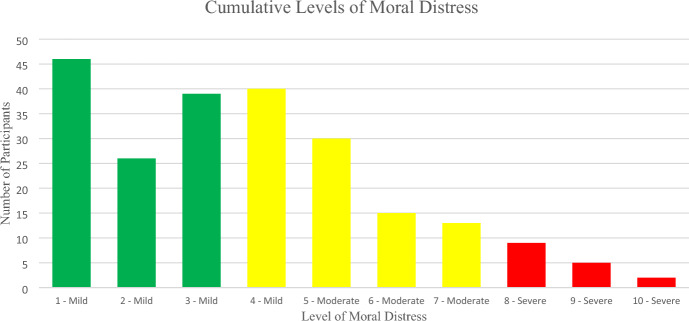
Moral distress levels: frequency of reported levels of moral distress

Overall, moral distress scores were mild (66.4%) to moderate (28.9%), with only a small fraction (4.7%) in the severe range (see Fig. 4). MDT scores for participants from ICUs were not different than MDT scores for participants from non-ICUs. Participants were able to identify multiple contributing factors for their moral distress, broken into five broad categories; clinical triggers, internal factors, external factors, legal and regulatory factors, and institutional or environmental factors. Clinical triggers contributed 27.0% to moral distress due in large part to concerns about current treatment and unclear goals of care. Communication factors contributed 26.3% to moral distress with inadequate team communication and being in the middle between key stakeholders contributing the most. Institutional and environmental factors accounted for 24.1% of the contributing factors with staff inexperience identified most often as the reason. Internal factors contributed to moral distress 17.1% of the time with feeling powerless and not wanting to cause harm were the reasons most often identified. Together, legal and regulatory, and “other” made up the remaining 3.5% of contributing factors to moral distress (see Table 2).

**Table 2:** Contributing factors to moral distress

Contributing factors	Totals		%
Clinical triggers	365	365	27.2
Concerns about current treatment (e.g., unnecessary, harm outweighs benefit, prolongs dying)	82		
Unclear goals of care	87		
Lack of consensus regarding treatment plan	53		
Inadequate symptom relief	50		
Lack of continuity in treatment plan or providers	46		
Plan is inconsistent with patient preferences	47		
Communication factors	349	349	26.0
Inadequate team communication	75		
Being in the middle between key stakeholders (e.g., between patient and family members, between members of the healthcare team and patients)	66		
Providers giving false hope	47		
Disrespectful behavior	47		
Conflict within the team	39		
Information withheld	32		
Conflict between the team and patient/family	29		
Incorrect information provided	14		
Internal factors	238	238	17.7
Feeling powerless	44		
Not wanting to cause harm	41		
Self-doubt, clinical inexperience	35		
Lack of assertiveness	23		
Professional values compromised	21		
Difficulty stating the ethical issues or ethical concerns	20		
Personal values compromised	20		
Fear of retribution	20		
Socialization to follow orders	14		
Legal and regulatory	36	36	2.7
Compromised care due to cost containment efforts	14		
Treatment plan focused on risk avoidance	11		
Tension between ethical & legal perspectives	9		
Other	2		
Institutional and environmental	323	323	24.1
Staff inexperience	60		
Inadequate resources to meet patients’ needs	48		
High turnover	48		
Lack of administrative support	45		
Lack of involvement in decision-making	28		
Tolerance of disruptive/abusive behavior	25		
Issues of unequal power within the healthcare system	22		
Work with clinically unsafe staff	15		
Policies that conflict with care needs	13		
Preferential treatment of some patients/families	12		
Compromised care to marginalized populations	7		
Other	30	30	2.3
Total counts of factors		1341	100

We attempted to obtain participants’ moral distress scores immediately pre and post participation in FEC however, not infrequently, participants had to leave the FEC abruptly to attend to patient care needs. Of the participants who provided data, participation in the first and second FECs resulted in a decrease in moral distress and the drop in moral distress was statistically significant after the second FEC. Participation in a third FEC had no statistically significant impact on levels of moral distress (See Table 3).

**Table 3:** Paired t-test comparing pre and post moral distress levels immediately before and after participation in a facilitated ethics conversation

FEC	N	Pre-FEC mean (CI)	Post-FEC mean (CI)	Difference in means (CI) post–pre	Paired T-test P-value
First FEC attended	38	3.28(2.57,3.98)	2.95(2.25,3.64)	− 0.28(-0.79,0.23)	0.2771
Second FEC attended	28	3.07(2.17,3.97)	2.59(1.74,3.45)	− 0.55(-1.01,-0.10)	0.0184*
Third FEC attended	10	2.00(0.93,3.07)	2.11(0.97,3.25)	0.11(-0.42,0.64)	0.6449

*The value indicates statistical significance for p < .05

#### Main Outcomes

When we examined moral distress scores for participants who stated they were thinking about leaving their current position due to moral distress, we could not detect a distinct pattern of sustained high levels of moral distress. The study design included a crossover trial with a two week “washout” period. The cross over design provided an opportunity to separate the influence of exposure to data alone from exposure to data with FEC. Due to the possibility of retained knowledge over time, linear modeling was performed to assess if there was any carryover effect. Statistical analysis for the hospital and peer sub scores of the Hospital Ethical Climate Survey showed no impact on moral distress from participation in the FEC. Before employing analytical methods, control groups were kept separated to keep the timing of the FEC intervention for groups A and B in the proper order. The remaining outcomes were assessed as a true crossover study design. Thus, the analysis consisted of two groups a control group who was not participating in FECs but was exposed to a posting of the average moral distress scores in the unit; and an intervention group who attended at least one FEC during the three weeks.

#### Control Group

Public posting alone of aggregate levels of moral distress with contributing factors had no statistically significant impact on levels of moral distress, moral agency, or perceptions about the work environment.

#### Facilitated Ethics Conversation (FEC) Intervention Group

Table 4 summarized the statistical analyses using study instruments. The results of the intervention were not all statistically significant however there were clear trends. Participation in FECs was consistent with a decrease in moral distress levels. The overall change in moral agency was not statistically significant, however, the difference between treatment groups A and B is significant. The paired t-test for the hospital sub scale of the HECS showed that participating in a FEC had a negative impact on levels of moral distress. However due to the carryover effect we performed mixed methods analyses and the statistical significance was not sustained.

**Table 4:** Statistical tables

Comparing levels of moral distress across time						
Group	Pre	Intermediate	Paired T-tests		Mixed modeling	
	Mean (CI)	Mean (CI)	Difference	P value	FEC-control	Adjusted P-value
Control Group B (Intermediate-Pre)	2.06(0.93,3.18)	2.61(1.40,3.82)	0.53(− 0.72,1.83)	0.3722	− 1.63(− 3.46,0.20)	0.0797
FEC Group A (Intermediate-Pre)	2.77(1.23,4.31)	1.69(0.16,3.22)	− 1.08(− 2.81,0.66)	0.2012		
	Intermediate	Post				
Control Group A (Post-Intermediate)	1.69(0.16,3.22)	1.38(0.41,2.36)	− 0.31(− 1.89,1.28)	0.6802	− 1.14(− 2.66,0.38)	0.1381
FEC Group B (Post-Intermediate)	2.61(1.40,3.82)	1.67(0.52,2.81)	− 0.944(− 2.11,0.22)	0.1048		
Comparing levels of moral activism across time						
Control Group B (Intermediate-Pre)	48.29(44.77,51	49.31(45.62,53	1.03(− 1.12,3.17)	0.3382	− 2.65(− 8.29,3.00)	0.3517
FEC Group A (Intermediate-Pre)	46.81(43.83,49	50.48(45.55,55	3.68(− 3.44,7.70)	0.0714		
	Intermediate	Post				
Control Group A (Post-Intermediate)	50.48(42.94,58	54.79(48.13,61	4.18(− 1.05,9.41)	0.106	4.66(− 0.19,9.52)	0.0595
FEC Group B (Post-Intermediate)	49.31(43.81,54	49.33(43.98,54	0.02(− 3.17,3.20)	0.9904		
Comparing levels of MHECS-peer subscale across time						
Control Group B (Intermediate-Pre)	4.50(4.37,4.63)	4.36(4.20,4.52)	− 0.14(− 0.29,0.01)	0.0719	− 0.25(− 0.56,0.05)	0.1027
FEC Group A (Intermediate-Pre)	4.56(4.43,4.69)	4.67(4.51,4.83)	0.11(− 0.02,0.25)	0.083		
	Intermediate	Post				
Control Group A (Post-Intermediate)	4.67(4.43,4.91)	4.58(4.35,4.82)	− 0.15(− 0.32,0.03)	0.0891	0.00(− 0.27,0.27)	0.9954
FEC Group B (Post-Intermediate)	4.36(4.12,4.60)	4.21(3.85,4.56)	− 0.15(− 0.39,0.08)	0.1864		
Comparing levels of MHECS-hospital subscale across time						
Control Group B (Intermediate-Pre)	3.60(3.33,3.87)	2.61(1.40,3.82)	− 0.79(− 0.93,-0.65)	< 0.0001	− 0.13(− 0.40,0.13)	0.3201
FEC Group A (Intermediate-Pre)	3.85(3.63,4.06)	3.19(2.97,3.41)	− 0.65(− 0.81,-0.49)	< 0.0001		
	Intermediate	Post				
Control Group A (Post-Intermediate)	3.19(2.86,3.53)	3.18(2.89,3.47)	− 0.02(− 0.25,0.20)	0.7909	0.02(− 0.13,0.17)	0.8012
FEC Group B (Post-Intermediate)	2.81(2.52,3.11)	2.83(2.49,3.18)	0.00(− 0.23,0.24)	0.9572		

#### Ancillary Findings

Participants for the study reported that it was “very important” to have an opportunity to discuss ethically challenging situations. Since participants valued the discussion about ethically challenging situations, it is important to note that pre/post intervention participation in discussions about ethically challenging situations increased significantly; observed discussions ((P < 0.0015), participated in discussions (P < 0.004) and initiated discussion (P < 0.007).

### Qualitative Outcomes

#### Control Group

Participants’ perceptions of score postings included five descriptive themes: increased awareness, increased conversation, call to action, normalization, and invasion of privacy. Table 5 provides a list of each theme, the definition of the theme, and a quote example from the narratives.

**Table 5:** Control group descriptive themes

Theme	Definition	Narrative quote
Increased awareness	Participant descriptions of increased awareness of moral distress and/ or its presence in self and/or others, and/or factors that may be contributing to moral distress, in response to posting of moral distress scores	“At the beginning they [scores] were lower, and as the study went on, they were getting higher, and I think that might be because people realized what moral distress was more than they thought they did in the beginning, and myself included.”
Increased conversation	Participant descriptions of increased conversation among the bedside team related to moral distress, its presence in self and/or others, and/or factors that may be contributing to moral distress, in response to posting of moral distress scores	“It brought out the conversation of staff to feel more comfortable talking about moral distress and why we have it and it’s okay people are listening, people do care."
Call to action	Participant descriptions of thoughts, conversations and/or actions prompted by the posting of moral distress scores, indicating a desire or plan for addressing moral distress in self and/or others, and/or the unit factors that may be contributing to moral distress among the care team	“I talked about it [scores] with one of our chaplains and was concerned about one shift and what people are rating themselves… they’re definitely feeling it. I was able to reach out and try to get some help with that for the staff."
Normalization	Participant descriptions indicating a sense of psychological relief or well-being from the realization others are experiencing similar feelings of moral distress and/or similar feelings related to the factors that may be contributing to moral distress, as related to the posting of moral distress scores	“We did have a few cases that were very distressing at the time. We were already kind of talking about it and then to find out that there were 10 and 12 people that felt the same way that you did kind of hit home.”
Invasion of privacy	Participant descriptions of curiosity and/or concern for potential privacy of colleagues related to outliers in extreme high and/or low moral distress scores in comparison to other scores	“There was one that marked severe and everyone was like we want to know who this person is who has such moral distress. Then I was like well, that kind of seemed like it backfired because in case you were that person you didn’t want to mark that again.”

#### Facilitated Ethics Conversations (FEC) Intervention Group

Interview participants were asked to describe their experience with participation in FECs and any impact to their moral distress. Participants generally described FECs using specific terms such as “beneficial”, “important”, “valuable”, “helpful”, and “productive”. Topics described by participants were consistent with known moral distress triggers including potentially inappropriate treatment (futility), disagreement concerning patient’s best interest, inadequate pain management, providing false hope and prolonged suffering at end-of-life (Hiler et al., 2018). Of the 20 participants, 18 described a decrease in their levels of moral distress after participation in FECs and 2 described feeling an initial increase in moral distress, in-part due to an increased awareness of ethical concerns and moral distress during study participation.

### Qualitative Themes

**Fig. 5:**
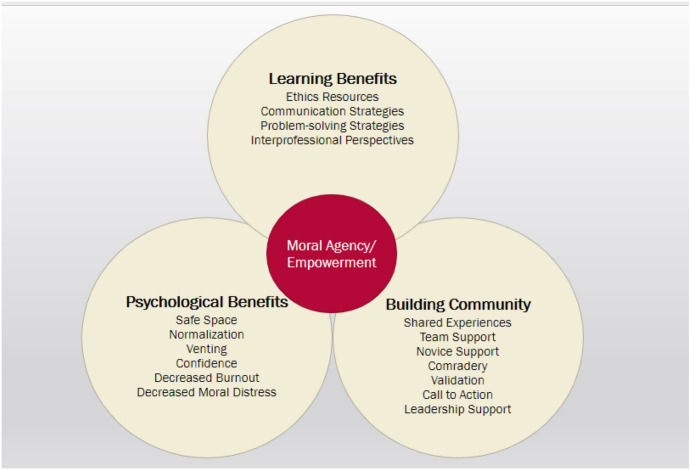
Visual diagram depicting the synergistic overlap of major descriptive, interpretive, and sub-themes

Three major themes emerged from participants’ perceptions of FECs including learning benefits, psychological benefits, and building community. Table 6 provides definitions of the major themes and lists the preceding sub-themes. The major themes appear to overlap in a synergistic way as some participants described feeling a sense of empowerment. This sense of empowerment is interpreted as moral agency. Figure 5 provides a visual diagram depicting the overlap of major themes that foster a sense of empowerment or moral agency.

**Table 6:** FEC Group descriptive and interpretive theme definitions

Theme	Definition	Sub Themes
Learning Benefits	Major descriptive theme that refers to any evidence from participant narratives indicating new knowledge and/or skills gained from attending FECs.	• Ethics Resources • Communication Strategies • Problem-solving Strategies • Interprofessional Perspectives
Psychological benefits	Major descriptive theme that refers to any evidence from participant narratives expressing relief from psychological stressors or increased feelings of emotional well-being associated with participation in FECs.	• Safe Space • Normalization • Venting • Confidence • Decreased Burnout • Decreased Moral Distress
Building Community	Refers to any evidence from participant narratives related to increased support, advocacy, communication, or trust within the practicing unit or team environment related to participation in FECs.	• Shared Experiences • Team Support • Novice Support • Comradery • Validation • Call to Action • Leadership Support
Increased Moral Agency	Major interpretive theme that refers to participant narratives that describe an ability to identify and deliberate ethical or moral concerns and feeling a sense of empowerment to take action after participation in FECs.	• Empowerment

### Learning Benefits

Participants described learnings from FECs including knowledge of ethical issues, ethics resources, strategies for addressing ethical concerns, and interprofessional perspectives. A skill-building effect was described in which learned communication and problem-solving strategies were utilized to help navigate moral distress and address ethical concerns at the bedside. An example of learning benefits is highlighted in the following quote:

Ethics Resources: “I wouldn’t be as afraid to make an ethics consult…I’ve been at this hospital for 20 years…in my mind I always thought of it as something sort of punitive. But it’s not. I don’t think I’m the only one that feels that way. Sometimes people think that an ethics consult means that what they’re doing is wrong. I think it would be good to make that clear to nurses and other practitioners. That it’s not anything punitive. It is just to help the patient and to advocate.”

Problem solving strategies: “I think that’s where the biggest impact is with a [FEC] is that you’ve been guided through the navigation of whatever your ethical dilemma was. Then the next time you have an ethical dilemma you’re able to think okay what is my problem? What am I seeing and what is the principal that I feel like is being challenged here? Is it about me, is it about the patient, is it about the team and then how can I resolve this?”.

Psychological Benefits: Participants described psychological benefits from FECs including a sense of normalization, validation and increased confidence to talk about their concerns. In addition, they described feeling decreased levels of moral distress and a sense of empowerment to take actions when ethical issues arise. An example of psychological benefit is highlighted in the following quote:

Confidence: “I am more confident in having those ethical conversations. I think that’s helped in giving me a lot more ideas on how to start the conversation and where to move forward and how to move forward with things.”

Decreased moral distress: “It’s wonderful to bring it to the forefront because then that opens other conversations. The more you talk about things like end of life and goals of care, the easier it gets for nurses to talk about it, the easier it gets for physicians to talk about it. The more we sort of normalize it then the easier it all becomes. Then ultimately your moral distress decreases.”

### Building Community

Sharing experiences during FECs seemed to foster a sense of comradery and community among unit team members. Participants described examples of other team members providing support for each other during FECs and in the work setting post conversations. Participants described a newly found sense of trust and community. A sense of comradery was evident in descriptions of the team’s shared approaches to creating solutions for addressing their moral distress and ethical concerns. Both novice and experienced clinicians found ethics discussions helpful for novice clinicians. During the interview process, some participants self-identified as novice clinicians and described feeling comfort in knowing that their more experienced colleagues experienced feelings of moral distress. Likewise, some participants self-identified as experienced clinicians and expressed a desire to support novice clinicians in finding ways to cope with moral distress. An example of this building community subtheme is highlighted in the following quote:

Novice support: “For me being the new nurse, when we have these discussions, it was great to see nurses who I look up to and respect on our unit to see them upset about some things. It just kind of reminds me okay they still struggle with certain things too, where this was still a really hard day for them, and this was a really hard patient outcome that happened that really affected them still. You think they’re older and they’re more experienced but being able to have that realization that other people are still having moral distress was really helpful especially for me being newer.”

Comradery: “We almost went overtime every time and other people would try and squeeze in and come into the meetings because they wanted to either share something or they wanted to be there for someone who was planning on mentioning something. I would try to plan my workday to go to this meeting to talk.”

Moral Agency: Participants described a sense of empowerment after participation in FECs that is interpreted as moral agency. Peter’s (2018) definition of moral agency is described as an ability to identify and deliberate ethical or moral concerns and feeling a sense of empowerment to act. A sense of empowerment after attending FECs was described by participants. Participant narratives generally contained more than one subtheme and the following quote provides an example of how the themes seem to overlap. In this quote example, the themes of learning benefit (communication, awareness), psychological benefit (confidence to speak up, feeling heard), and community building (sharing, support) provided a sense of empowerment to take action (moral agent).

Empowerment: “When you become more clear as to what it is [moral distress], you see it happening. A growing awareness or understanding of what it is, beginning to identify it in your surroundings and then the sense of empowerment like someone is going to listen to me. I don’t need to be afraid to talk about it or think nobody cares. I can go forward with this situation and share it with someone knowing that I’m going to be heard. Then I feel empowered”

Unexpected findings: Participant’s perceptions of the importance of facilitator skill was a major and unexpected finding in this study. The interviews did not include questions related to the facilitator or facilitation skills. All FECs were conducted by the first author (LDW) who has expertise in clinical ethics, moral distress, and group facilitation. Participants identified the skill of the facilitator as critical to the success of FECs. Important facilitator skills described by participants included expertise concerning ethical issues, ability to ask open-ended questions, skill in guiding the discussion, ability to draw out participant concerns, and skill in facilitating team engagement in exploring strategies. An example of participant perception of facilitator skill is provided in the following quote:

Facilitator Skill: “I think the quality of the facilitator can’t be underestimated... she has the highest level of ability to ask a simple question to draw out issues. I think that the success of our [conversations] may have somewhat been affected by the quality of her work. She just knows how to draw things right out of people.”

## Discussion

This study used both quantitative and qualitative methods to evaluate the impact of an intervention to address moral distress. The study demonstrates the feasibility of tracking and responding to moral distress in real time. Simply posting aggregate scores for moral distress and identifying contributing factors had no statistically significant impact on participants’ level of moral distress. However, posting aggregate scores raised awareness and prompted conversation among the team concerning moral distress. The measuring and tracking of unit-based moral distress scores may be an effective way for leaders to normalize the experience of moral distress, assess levels of distress, build awareness, and promote conversation about moral distress. However, as an intervention score postings were insufficient to navigate the complexities of the experience of moral distress. The finding that we could not detect a discernable pattern of sustained high levels of moral distress for participants who stated they were thinking about leaving their current position due to moral distress suggests there is a need for a more sophisticated way to link levels of moral distress with being at risk for leaving a position.

While quantitative evidence supporting the FECs as an intervention for addressing moral distress may not have reached overall statistical significance, the trend was favorable. We found statistically significant difference in moral agency between treatment groups A and B which suggests that the impact of attending FECs may not be immediately detectable, or that moral agency takes time to develop once exposed to the intervention, consistent with interventions aimed at moral empowerment (Abbasi et al., 2019). The statistically significant difference from pre to post interventions in self-report of observation of, participation in, and initiation of conversations around ethically challenging situations demonstrates support for the positive impact FECs can have on a work environment.

FECs by their nature are unstructured and informal. The content is driven by participants who may want to discuss a particularly challenging case, or more general topics. This informal structure may explain in part why moral distress scores did not show a statistically significant difference. Given that one of the most frequently identified contributing factors to moral distress was a clinical trigger, and the discussion may not have been in close proximity to the trigger, it makes sense that the discussion may not be linked closely enough to the triggering event.

Qualitative evidence clearly supports FECs as a meaningful intervention to address moral distress, a finding consistent with prior research (Chiafery et al., 2018; Janssens et al., 2015; Hamric & Epstein, 2017). The strength of the qualitative data analysis underscores the message from the American Statistical Association (Wasserstein et al., 2019), namely that statistical significance is not the same as importance. Fixating on the “p < 0.05” as the criterion to indicate whether an intervention is important may be misleading and certainly does not tell the whole story (Hayat et al., 2019).

The qualitative data analysis revealed a conceptual framework of major themes and subthemes that offer insights for potential targeted strategies for moral distress interventions. Examined in their entirety, the themes suggest FECs are an effective strategy to decrease moral distress and perhaps more importantly, enhance moral agency. It suggests that changing the moral distress score may be less important than having an opportunity to discuss and explore the experience of moral distress. The concept of moral community emerged in the participant narratives describing the importance of trust in leadership to promote an ethical work environment. The benefits derived from participation in FECs may be tied to the creation of a broader moral community which fosters a culture of openness and trust and promotes personal integrity, moral agency, and empowerment (Hamric & Wocial, 2016; Traudt Liaschenko, & Peden-McAlpine, 2016; Liaschenko & Peter, 2016). Programs such as FECs can address feelings of moral distress and by doing so may serve as a retention strategy by nurturing a strong moral community.

Consistent with prior research (Abbasi et al. 2021; Browning, 2013; Robinson et al., 2014; Liaschenko & Peden-McAlpine, 2016), this study demonstrates an inverse relationship between moral distress and moral agency, namely lower levels of moral distress are associated with higher levels of moral agency. FECs allow HCP to engage in moral discourse and represent a meaningful ethics resource that can support HCPs experiencing high levels of moral distress. Systematic reviews of published moral distress intervention research studies and the recent report from the United States Surgeon General (Murthy, 2022) underscore organizations’ responsibility to provide opportunities for collective engagement in deliberations involving moral issues that arise in clinical practice (Amos & Epstein, 2022; Caram et al., 2022). FECs are one potential way institutions can meet this obligation.

While the details varied, the three most frequently identified contributing factors to levels of moral distress were clinical triggers, communication, and institutional/environmental constraints, which is consistent with other published studies examining moral distress (Houston et al., 2013; Wilson et al., 2013; Atashzadeh-Shoorideh et al., 2021; Austin et al., 2017; Ulrich et al., 2019; Musto & Rodney, 2018). FECs may help participants explore these contributing factors, however with mostly nurses participating in the FECs, there would be little opportunity to engage other members of the healthcare team in meaningful dialogue about how to address these factors. The strongest contributors to moral distress for this study support previous observations that an interprofessional FECs may have a greater impact on moral distress (Berger et al., 2019).

The complexity of the construct of moral distress will continue to hinder efforts to measure interventions to address it. The ongoing debate in the literature about the definition of moral distress reinforces the idea that not all distress is moral distress. However, all distress deserves attention. Whether or not it is moral (as in a matter of professional integrity or the constraints are real not just perceived) can only be determined if the individual experiencing it explores the ethical complexity driving the feelings of distress. When an individual claims moral distress, we cannot accept it at face value. However, when an individual claims moral distress, we must respect that judgment until an exploration can provide more clarity. Moral distress is based in part on the individual’s judgment about the morality of a situation. A FEC is one way to expose other perspectives and ultimately influence the judgment. The findings related to facilitator skill provide insight for developing curriculum to train qualified facilitators and reinforce the importance of facilitator skill in leading FECs Delaney et al. (2021).

### Limitation

This study had limitations, notably the low response rate and attrition of participants over the duration of the study, a challenge faced by many who endeavor to conduct intervention studies to address moral distress (Amos & Epstein, 2022). The selected study instruments and manipulation of those instruments may explain in part the lack of statistical significance. Simplifying the instruments to reduce participant response burden may have compromised instrument validity and reduced instrument sensitivity to detect variability in the levels of moral distress or moral agency.

Perhaps this study poses more questions than the ones answered. We did not have enough participants to achieve the necessary power for the study to identify how much of a change on the MDT would be enough to say with confidence that the change was meaningful. For example, while there was not a clear statistically significant drop in moral distress, we still do not know how much of a drop is important to the person experiencing it, or if it is most important to address moral distress levels when they are high rather than offer interventions when moral distress is at a low or moderate level.

## Conclusion

The multi-method approach used for this intervention study enhances the strength of conclusions. Tracking and responding to moral distress in real time and measuring the impact of an intervention to address moral distress is feasible. Participants in FECs described decreased moral distress and enhanced moral agency through a combination of learning benefits, psychological benefits, and building community. FECs have the potential to lower moral distress, promote moral agency, and enhance a work environment that is open to discussion about ethically challenging situations. More research exploring the relationship between moral distress and moral agency, and interventions to decrease moral distress and increase moral agency is needed.

## Data Availability

Data will be made available to the editors and publishers of the journal upon request.

## Appendix A: Qualitative Interview Questions and Prompts

1. Tell me your impressions about the posting of moral distress scores and resources on your unit.

Prompt:

- What was your perception of the moral distress scores and list of resources?
- Tell me about any conversations you had concerning the moral distress scores and list of resources.
- Did you access any of the resources listed? If so what resources?

2. What impact did the scores and resource postings have on your moral distress?
3. Describe your experience with participation in FECs.

Prompt:

- Tell me what happened in the FECs?

How did it go?

What kind of topics came up?

4. What impact did participation in the FECs have on your moral distress?
5. What impact do you think either intervention will have on how you navigate moral distress in the future?
6. What haven’t we talked about with regards to your participation in this project in general that you would like to share?

## Code Availability

No software was used for the qualitative portion of this research.
