# Correction to: Evaluation of Interventions to Address Moral Distress: A Multi-method Approach

**DOI:** 10.1007/s10730-023-09512-3

**Published:** 2023-08-09

**Authors:** Lucia D. Wocial, Genina Miller, Kianna Montz, Michelle LaPradd, James E. Slaven

**Affiliations:** 1 Medstar Washington Hospital Center, John J. Lynch, MD Center for Ethics, 110 Irving Street, NW EB 310, Washington, DC 20002 USA; 2 Fairbanks Center for Medical Ethics, Charles Warren, Indianapolis, IN USA; 3 Indiana University Health, Indianapolis, IN USA; 4 Syneos Health, Morrisville, NC USA; 5 Department of Biostatistics and Health Data Science, Indiana University, Bloomington, IN USA

**Correction to: HEC Forum**

10.1007/s10730-023-09508-z.

In the original publication of the article, the abbreviation UBECs appearing in table 6 should be FECs. The correct version of table 6 is provided in this correction.

The original article has been corrected.

**Table 6:** FEC Group descriptive and interpretive theme definitions

Theme	Definition	Sub Themes
Learning Benefits	Major descriptive theme that refers to any evidence from participant narratives indicating new knowledge and/or skills gained from attending FECs.	• Ethics Resources • Communication Strategies • Problem-solving Strategies • Interprofessional Perspectives
Psychological benefits	Major descriptive theme that refers to any evidence from participant narratives expressing relief from psychological stressors or increased feelings of emotional well-being associated with participation in FECs.	• Safe Space • Normalization • Venting • Confidence • Decreased Burnout • Decreased Moral Distress
Building Community	Refers to any evidence from participant narratives related to increased support, advocacy, communication, or trust within the practicing unit or team environment related to participation in FECs.	• Shared Experiences • Team Support • Novice Support • Comradery • Validation • Call to Action • Leadership Support
Increased Moral Agency	Major interpretive theme that refers to participant narratives that describe an ability to identify and deliberate ethical or moral concerns and feeling a sense of empowerment to take action after participation in FECs.	• Empowerment

